# Effects of Different Types of High-Intensity Interval Training (HIIT) on Physical Performance in Female Basketball Players—A Systematic Review

**DOI:** 10.3390/life15081180

**Published:** 2025-07-25

**Authors:** Ilma Čaprić, Mima Stanković, Ivana Bojić, Borko Katanić, Igor Jelaska, Luka Pezelj, Bojan Masanovic, Valentina Stefanica, Karuppasamy Govindasamy

**Affiliations:** 1 Department of Biomedical Sciences, State University of Novi Pazar, 36300 Novi Pazar, Serbia; icapric@np.ac.rs; 2 Faculty of Sport and Physical Education, University of Niš, 18000 Niš, Serbia; bojicka2003@yahoo.com; 3 Montenegrin Sports Academy, 81000 Podgorica, Montenegro; borkok@ucg.ac.mn; 4 Faculty of Kinesiology, University of Split, 21000 Split, Croatia; jelaska@kifst.hr; 5 Faculty of Maritime Studies, University of Split, 21000 Split, Croatia; luka.pezelj@pfst.hr; 6 Faculty for Sport and Physical Education, University of Montenegro, 81400 Niksic, Montenegro; bojanma@ucg.ac.me; 7 Department of Physical Education and Sport, Faculty of Sciences, Physical Education and Informatics, National University of Science and Technology Politehnica Bucharest, Pitesti University Center, 060042 Pitesti, Romania; 8 Department of Sports, Recreation and Wellness, Symbiosis International (Deemed University), Hyderabad Campus, Modallaguda (V), Nandigama (M), Rangareddy, Hyderabad 509217, India; govindasamy.k@siu.edu.in

**Keywords:** speed, aerobic capacity, body composition, strength, repeated speed, VO_2max_

## Abstract

The aim of this systematic review was to examine the effects of high-intensity interval training (HIIT) on physical performance and body composition in female basketball players. The review followed PRISMA guidelines, and the protocol was registered in the PROSPERO database (registration number: CRD420251006285). A comprehensive search was conducted across PubMed, Scopus, Web of Science, and Google Scholar. Nine studies that met the inclusion criteria were analyzed, with intervention durations ranging from 4 to 12 weeks. Despite differences in protocols, a majority of studies reported improvements in VO_2max_ (6/9), explosive strength (7/9), agility (5/6), and speed (5/6) and reductions in body mass and fat percentage (3/3). These findings highlight HIIT as an effective method for enhancing both aerobic and anaerobic capacities, as well as optimizing body composition. Despite variations in study protocols, HIIT consistently offers improvements in performance, irrespective of training level. The results underscore the importance of HIIT in preparing athletes, not only during the preseason but also throughout the competition period. Coaches should consider integrating HIIT into training programs, adjusting intensity and volume based on the season to optimize performance and prevent overtraining.

## 1. Introduction

Basketball, one of the most popular sports worldwide that attracts millions of fans globally [1], has undergone significant evolution throughout its history in terms of game rules, technique, and tactics, becoming a dynamic and high-intensity sport [2]. Achieving high performance in basketball requires the development of a broad spectrum of abilities, including motor, functional, cognitive, and conative skills [3,4]. Explosive movements such as jumping, sprinting, and dribbling play a critical role in performance and demand high levels of strength, agility, and speed [5]. This structure of the game enables athletes to repeatedly perform high-intensity efforts while maintaining optimal performance throughout the match [6,7].

Female athletes, including basketball players, face distinct physiological challenges compared with their male counterparts [8]. These challenges include differences in body composition, such as higher body fat percentage, as well as hormonal fluctuations that may influence training responses [9]. As a result, female athletes may respond differently to interventions such as high-intensity interval training (HIIT). While HIIT is well documented in enhancing performance among male athletes, research on its effects in female populations remains limited, accounting for only 20% of all sports science studies [10]. In addition, HIIT enhances physical performance by stimulating fast-twitch muscle fibers, which are recruited during high-intensity efforts. This neuromuscular activation promotes the secretion of anabolic hormones such as testosterone and growth hormone. Consequently, HIIT contributes to significant improvements in muscular strength and power, particularly among trained athletes [11]. HIIT involves short bouts of intense physical exertion interspersed with periods of rest or low-intensity active recovery, making it highly effective in improving both aerobic and anaerobic endurance in female basketball players [12]. In line with adaptation models, short HIIT intervals provide variable volume and intensity, offering flexibility in training load to enhance adaptation. In contrast, long-interval HIIT optimizes the anaerobic system and neuromuscular load, focusing on specific physiological systems [13]. Beyond endurance, HIIT contributes to the development of strength, explosiveness, and agility—key attributes for success in basketball, which involves frequent directional changes, jumps, and sprints [14]. This training approach can be adapted to the specific physiological characteristics of female athletes, including hormonal cycles and anthropometric differences, thereby providing optimal conditions for performance enhancement and injury prevention [15].

Systematic reviews and meta-analyses have demonstrated that HIIT improves VO_2max_, anaerobic performance, and repeated sprint ability (RSA) in male athletes, while also requiring less training time than traditional methods [16,17]. However, these findings are primarily based on studies with male participants, leaving a considerable gap in the understanding of HIIT’s impact on women. Moreover, studies involving female athletes are often constrained by the influence of hormonal cycles, which can alter training responses [18]. Given that female athletes have specific hormonal profiles and that the menstrual cycle affects their physiological status, it is essential to consider these factors in training design to achieve peak performance [19].

In basketball, monitoring player workloads during the preseason and competitive season is crucial for strategically managing training loads and promoting physical adaptation [20,21]. Fatigue, reduced protein catabolism, and impaired lipolysis can hinder energy mobilization and negatively affect performance [22]. Stress-induced increases in cortisol and decreases in testosterone may further reduce shooting ability and muscle recovery [23,24]. Although hormonal responses in female basketball players are under-researched, additional investigations are warranted [10].

Despite growing interest in women’s basketball and other team sports, there remains a need for a deeper understanding of how HIIT can be specifically tailored to optimize performance in female athletes. Although previous reviews and meta-analyses have predominantly focused on male team-sport athletes, there is a lack of sufficient results to draw conclusions about the effects of HIIT on female team-sport athletes, as studies involving female participants account for only 20% of all sports and exercise science research [25]. In addition, while a recent systematic review with meta-analysis investigated the effects of HIIT on basketball players in general, the current study focused on the female population, which is still underrepresented in the sports science literature, to better understand the effects of HIIT in this particular population [26]. Differences in fatigue rates, anthropometric features, and neuromuscular adaptations between men and women suggest that HIIT protocols designed for male athletes may not be directly applicable to women [27]. This highlights the necessity for further investigation into HIIT approaches that account for the unique needs of female athletes, particularly in team sports like basketball, where strength and agility are paramount for success [5]. This study aims to emphasize the current lack of a comprehensive review specifically addressing the effects of various types of HIIT on body composition, aerobic and anaerobic performance, and physical fitness in female basketball players. Although HIIT is widely used in athletic training across numerous disciplines, there is a scarcity of research focused on female basketball players, who have distinct requirements in terms of physical conditioning, agility, strength, and endurance.

This review provides a novel perspective by focusing on this specific athletic population and analyzing how different HIIT protocols influence body composition, aerobic and anaerobic capacity, and physical fitness in female basketball players. By examining these elements, the study aims to contribute to a better understanding of how specific HIIT interventions can enhance physical performance in basketball, offering new insights for training optimization and injury prevention in female athletes. Accordingly, the objective of this review is to analyze the effects of different HIIT protocols on key physical parameters such as body composition, aerobic capacity, anaerobic performance, and muscular strength in female basketball players. This review seeks to address the existing research gaps and to provide practical insights into how HIIT can be adapted to the needs of female athletes, thereby optimizing their performance on the court.

## 2. Materials and Methods

### 2.1. Literature Identification

The PROSPERO registration number for this review is CRD420251006285. The search and analysis of studies were conducted in accordance with PRISMA guidelines [28]. The review included studies published between 2015 and March 2025. A comprehensive literature search was carried out using the following databases: Google Scholar, PubMed, Web of Science, Cochrane Library, ProQuest, and ScienceDirect.

The search strategy targeted studies related to HIIT and female basketball players, employing the following keywords and combinations, either individually or in conjunction: (“HIIT” OR “high-intensity interval training”) AND (“women” OR “female”) AND (“basketball” OR “female basketball players”) AND (“physical performance” OR “body composition” OR “aerobic fitness” OR “anaerobic fitness” OR “muscle fitness” OR “maximal oxygen uptake” OR “VO_2max_” OR “repeated sprint ability” OR “agility” OR “speed” OR “explosive power” OR “explosive strength”). Literature screening, quality assessment, and data extraction were performed in conjunction with a review of the reference lists of the included studies. Following a mutual evaluation, studies were either excluded or selected for further analysis.

Two independent reviewers (I.Č. and M.S.) conducted the literature search; identified relevant studies; and performed selection, quality assessment, and data extraction. The screening process began with a review of titles, followed by abstracts and then full-text articles to assess their eligibility for inclusion. Studies that did not meet the systematic review criteria were excluded. Additionally, the reference lists of selected articles were manually searched to identify potentially relevant studies. In the event of discrepancies between reviewers, disagreements were resolved through consensus or, if necessary, with the involvement of a third reviewer. If full-text articles were not available, the authors were contacted directly via email.

### 2.2. Eligibility Criteria

Study Design and Participants: The PICOS framework was used to determine inclusion criteria (Table 1) including randomized controlled trials (RCTs) investigating HIIT interventions with no restriction regarding the date of publication. The search was limited to studies published in English. Eligible participants were female basketball players competing at elite, sub-elite, or collegiate levels. Baseline fitness level and training experience were not criteria for inclusion. Studies involving recreational female athletes or mixed-gender samples without sex-specific data were excluded.

Type of Intervention: Training programs were required to last a minimum of two weeks and to include at least one experimental group. The exercise intensity had to be between 80 and 100% of maximal heart rate (HRmax). The number of weekly training sessions was not an inclusion criterion. Studies that combined HIIT with other forms of training that could potentially influence the overall outcomes were excluded.

Types of Measurable Outcomes: The primary outcomes for this systematic review were VO_2max_, RSA, change-of-direction speed, linear speed, explosive power, and body composition.

### 2.3. Data Extraction

Data were extracted independently by two researchers, while a third author performed a cross-check to verify the accuracy and comprehensiveness of the extracted information. Any discrepancies were resolved through consensus, with the involvement of a third and, when necessary, a fourth reviewer. Following this process, the data were entered into an Excel spreadsheet.

Data extraction was conducted in accordance with a standardized protocol recommended by the Cochrane Consumer and Communication Review Group [29]. The extracted information included the following study characteristics: authors, title, and year of publication; participant data (including sample size, age group, and sex); and detailed information on the interventions (such as intensity, duration, and frequency of training sessions). Additionally, data on study outcomes were collected, focusing on physical performance variables including VO_2max_, repeated sprint ability, change-of-direction speed, linear speed, explosive strength, and body composition. The reviewers were not blinded to the authors, affiliations, or journals in which the studies were published.

## 3. Results

### 3.1. Study Selection and Characteristics

The electronic database search and reference screening of retrieved articles initially yielded a total of 9580 potentially relevant studies. Of these, 826 were excluded as duplicates. Subsequently, 8754 studies were screened based on titles and abstracts, resulting in the exclusion of 7976 studies that did not meet the inclusion criteria.

**Figure 1:**
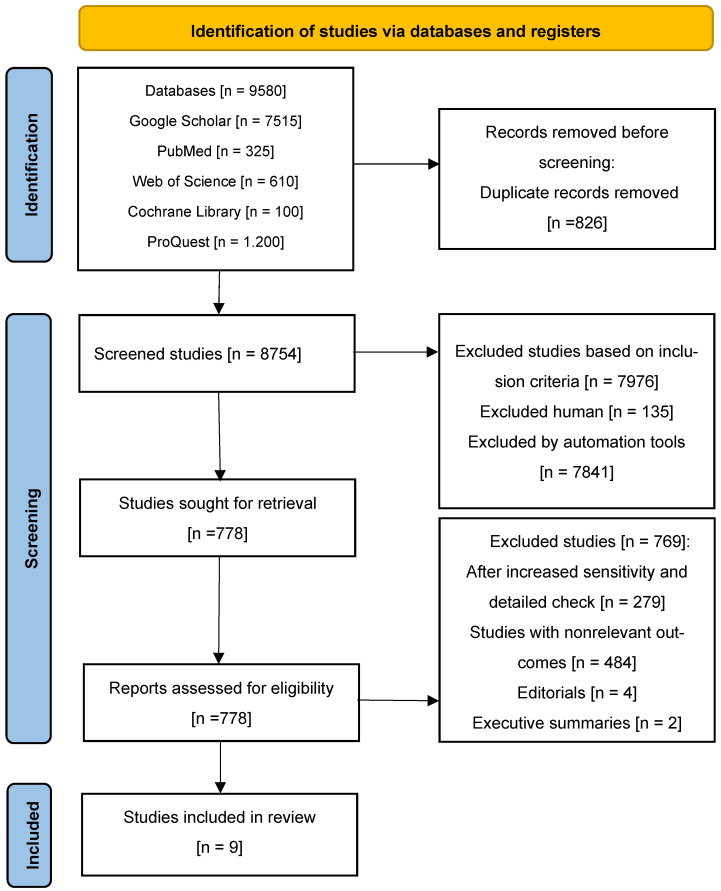
Process of identifying studies for the systematic review.

A total of 778 full-text articles were then assessed for eligibility. After detailed evaluation, 769 studies were excluded due to having irrelevant outcomes, being editorial in nature, or being executive summaries. Ultimately, nine full-text studies were included in the final systematic review (Figure 1).

### 3.2. Study Quality

The methodological quality of the studies was evaluated in accordance with PRISMA criteria using the PEDro scale [30]. The PEDro scale scores studies from 0 to 11, with cut-off points defined as follows: 0–3 indicating low methodological quality, 4–5 moderate quality, 6–8 good quality, and 9–11 excellent quality. Two independent reviewers (I.Č. and M.S) evaluated methodological quality using suitable checklists. Cohen’s kappa coefficient was used to estimate inter-rater agreement for full-text eligibility and methodological quality assessment. In cases of disagreement, a third reviewer checked the data before making the final conclusion. The inter-rater agreement was excellent, with a kappa coefficient of 0.92, see Table 2 and Table 3.

Each study was carefully analyzed and coded according to the relevant descriptive variables, including participants’ age, sample size, study duration, type of control group [no exercise, regular training], specific details related to the exercise regimen and intensity of the control group, as well as the mode and intensity of the experimental group’s exercise. Additionally, data were coded regarding the ratio between exercise and rest periods, and the relationship between the duration of the intervention and its effect on study outcomes. These variables encompass key factors that may influence results, including exercise specificity, physical characteristics of participants, and the length and structure of interventions, enabling a more detailed analysis of factors that may contribute to or limit the effects of interventions in each study.

All studies that met the inclusion criteria were original scientific articles published in English between January 2015 and June 2025. The total number of participants was 179, with the highest number of participants in the studies by Aschendorf et al. [33], Mourgan et al. [37], and Haghighi et al. [38] and the lowest in the study by Sanchez-Sanchez et al. [32]. Participant age ranged from 14 to 23 years. A total of eight studies used HIIT for the experimental treatment [31,32,33,35,36,37,38,39], and one study used a combination of two different HIIT training protocols [34]. HIIT training included tests to assess body composition (BF, FFM), muscular fitness (V-cut test, RSA, CMJ, CMJa, SJ, VJ, MAT, SAT), with the majority of tests aimed at assessing anaerobic and aerobic fitness (VO_2max_ VT, 3000-m race, PPO, MPO, VIFT, YYIR1, COD Shuttle, run 20m, RPE, 30-15 IFT, Shuttle, 30-15 VIFT, RSA).

### 3.3. Effects of HIIT on Body Composition

Several studies investigated the impact of HIIT on body composition in female basketball players and recorded improvements in various variables. BF (body fat) was reduced by 5–15% [31,35], while FFM (fat-free mass) increased by 5–10% [34,36]. BMI (body mass index) showed reductions of 2–5% [32,38].

### 3.4. Effects of HIIT on Aerobic and Anaerobic Capacity

All studies analyzing the impact of HIIT on the physical performance of female basketball players showed significant improvements in various variables. Increases in VO_2max_ ranged from 12% to 25% [31,34,39] while anaerobic capacity increased by 15% to 40% [32,35,39]. Peak power output (PPO) improved by 10–15% [33,34], and performance in the 3000 m race by 5–10% [31]. Yo-Yo IR1 test improved by 26.7% indicating a very large effect [39]. Results in tests such as 30-15 IFT, Shuttle Run 20m, and RSA recorded increases of 10–20% [31,34,36]. Additionally, power (VIFT) increased by 5–10% [38], while parameters such as VCO2 and VEGF showed improvements in oxygen consumption efficiency and vascular response, with changes ranging from 2% to 15% [35,37].

### 3.5. Effects of HIIT on Physical Fitness

In studies that examined the impact of HIIT on the physical performance of female basketball players, improvements were noted in various variables. The V-cut test improved by 10–15% [32], while results in CMJ (countermovement jump) and CMJa showed strength increases of 5–10% [35,38]. SJ (squat jump) improved by 10–15% [32,33], while MAT (maximum agility yest) results increased by 5–10% [31]. SAT (shuttle agility test) demonstrated agility improvements of 10–20% [37], while *T* test showed improvements of 3.7% [39]. Also, VJ (vertical jump) increased by 5–13.5% [38,39]. Speed in tests such as the 10m and 20m sprint, as well as change of direction (COD), improved by 4–15% [32,35,39].

## 4. Discussion

This systematic review demonstrates that HIIT protocols effectively enhance physical performance in female basketball players, improving key conditioning parameters such as aerobic endurance, strength, and explosiveness, thereby contributing to overall sports efficiency and on-court performance.

HIIT represents an alternative training modality for developing aerobic conditioning in team-sport athletes, aiming to closely replicate game-specific demands and characteristics. HIIT enhances neuromuscular coordination and sprint-specific movement patterns [40], augments anaerobic capacity, and enables athletes to sustain high-intensity efforts, which are critical in basketball contests [41]. It also conditions the body to recover rapidly between efforts [42], which is essential for repeated sprints [43]. Although HIIT has proven effective for enhancing linear and repeated sprint performance [44], interventions employing VIFT did not yield significant improvements in RSA [35,45]. VIFT primarily targets aerobic conditioning but lacks sufficient intensity to elicit the neuromuscular adaptations required for RSA enhancement [46,47]. In contrast, dedicated RSA training protocols result in significant RSA improvements [48]. Moreover, a shorter four-week VIFT regimen did not produce notable enhancements in 20 m sprint performance, whereas six-week HIIT protocols achieved superior outcomes [34,46]. Consequently, HIIT programs frequently incorporate repeated sprint components alongside technical–tactical training.

Earlier investigations have compared HIIT with other training strategies in female basketball players, such as small-sided games [49] or HIIT with one or three changes of direction (CODs) [32]. Rodríguez-Fernández et al. [35] constituted the first study to evaluate the differential effects of two HIIT modalities (i.e., C-HIIT versus N-HIIT). Their primary findings indicate that both C-HIIT and N-HIIT protocols enhanced aerobic performance (30-15 VIFT) after six weeks (8.5% and 3.3%, respectively), while only the C-HIIT protocol yielded a 3.8% improvement after three weeks.

VO_2max_ is considered the most valid indicator of cardiorespiratory endurance and aerobic fitness. Recent evidence suggests that HIIT can elevate VO_2max_ by 14% to 28% [31]. Additionally, Mourgan et al. [37] reported that five weeks of HIIT elicited significant increases in VO_2max_ (5.6%) and VCO_2_ (8.4%) in young female athletes. Other studies demonstrate that both HIIT and continuous moderate-intensity training similarly improve VO_2max_ in female basketball players, with increases ranging from 5% to 14% [50,51,52,53].

The implementation of ten basketball-specific HIIT sessions over five weeks has been shown to augment aerobic performance in young female basketball players. The earlier literature suggested that endurance training might attenuate strength parameters [54]; however, recent findings [33] contradict this, as the HIIT cohort exhibited improvements in sprint times and ball-passing accuracy, while vertical and horizontal jump performances remained unchanged. These outcomes coincide with observations from other investigations [55,56,57]. Furthermore, Mourgan et al. [37] and Haghighi et al. [38] underscored the efficacy of HIIT in enhancing jump-specific strength, thereby improving explosiveness and vertical jump performance in young female basketball players.

Moreover, the HIIT group achieved a 26.5% improvement in Yo-Yo test distance, consistent with results observed in other female team sports. For example, volleyball athletes demonstrated a 17% Yo-Yo improvement following a four-week HIIT intervention [58], while handball players exhibited a 17% increase after 16 HIIT sessions. Aschendorf et al. [33] also reported Yo-Yo enhancements akin to those achieved through small-sided games across various sports disciplines [45,59,60].

Previous reviews have concluded that HIIT constitutes an effective conditioning strategy that significantly enhances physical performance across multiple sports [44,61]. Stanković et al. [61] established that HIIT exerts a substantial impact on VO_2max_, repeated sprint ability, change-of-direction speed, linear sprint speed, and explosive lower-body strength in female team-sport athletes, irrespective of competitive level. Nevertheless, interventions shorter than three weeks often fail to produce significant VO_2max_ improvements, as exemplified by Burgomaster et al. [51], owing to variations in training modality and intensity.

The endocrine response to HIIT in female athletes is complex, influenced by menstrual cycle phase, psychological stress, and training load [62]. Prolonged or intense seasonal workloads may disrupt hormonal homeostasis and adversely affect performance [63]. Consequently, regular monitoring of cortisol and testosterone levels may facilitate stress management, recovery optimization, and injury prevention.

Aschendorf et al. [33] reported no changes in body fat composition following HIIT in female basketball players, contrasting with findings in handball athletes, wherein Alonso-Fernández et al. [64] observed a 3.4% reduction in body fat post-HIIT. Moreover, it should be noted that there is very little research addressing the impact of HIIT on body composition in women, particularly in the context of team sports. This highlights the need for further studies focusing on different aspects of HIIT training to better understand its potential impact on women’s body composition and to identify key factors that may contribute to its effectiveness or inefficiency in this context. Future research should explore the biological, psychological, and hormonal aspects that influence the impact of HIIT in female athletes to have a deeper understanding.

## 5. Practical Applications

The findings of the present review suggest that HIIT programs, regardless of type, lead to improvements in VO_2max_, RSA, change-of-direction speed, speed, explosive lower-body strength, and body composition in female basketball players engaged in team sports. Regardless of training level or competitive experience, HIIT provides benefits both in the preparatory period, when physical abilities are elevated to a higher level, and in the competitive period, where these abilities can be maintained. It is important for coaches to utilize HIIT methods in preparing their teams and to adjust the type of HIIT training depending on the season in which it is applied.

## 6. Limitations

Although this systematic review provides important insights of the impact of HIIT training on physical performance in female basketball players, there are significant limitations to the existing literature. First, HIIT regimens vary significantly between studies, making a direct comparison and generalization of outcomes challenging. Secondly, many studies have small sample sizes, which might impact the statistical power and reliability of the findings. Furthermore, there has been little research of the effect of HIIT on hormonal status and body composition in women participating in team sports. Furthermore, some studies compare two types of HIIT but lack a control group, which makes it difficult to draw definitive conclusions about the effectiveness of specific protocols. More research is needed to thoroughly investigate optimal protocols, individual variations, and periodization of HIIT in female athletes.

## Figures and Tables

**Table 1:** Description of the PICOS strategy.

PICOS	Inclusion Criteria
Population	Female basketball players competing at elite, sub-elite, or collegiate levels
Intervention	HIIT programs lasting at least 2 weeks with exercise intensity 80–100% HRmax
Comparison	Control group or other training interventions (when applicable)
Outcome	VO_2max_, RSA, change-of-direction speed, linear speed, explosive power, body composition
Study Design	Longitudinal, randomized, controlled trials; no date restriction

**Table 2:** PEDro scale results.

Study:	1	2	3	4	5	6	7	8	9	10	11	∑
Maha et al. (2015) [31]	Y	Y	N	Y	N	N	N	Y	Y	Y	Y	7
Sanchez-Sanchez et al. (2018) [32]	Y	Y	Y	Y	N	N	N	Y	Y	Y	Y	7
Aschendorf et al. (2019) [33]	Y	Y	N	Y	N	N	N	Y	Y	Y	Y	6
Zeng et al. (2022) [34]	Y	Y	Y	Y	N	N	N	Y	Y	Y	Y	7
Rodríguez-Fernández et al. (2023) [35]	Y	Y	Y	Y	N	N	N	Y	Y	Y	Y	7
Apaydın et al. (2023) [36]	Y	Y	N	Y	N	N	N	Y	Y	Y	Y	6
Mourgan et al. (2024) [37]	Y	Y	Y	Y	N	N	N	Y	Y	Y	Y	7
Haghighi et al. (2024) [38]	Y	Y	N	Y	N	N	N	Y	Y	Y	Y	6
Fang et al. (2024) [39]	Y	Y	Y	Y	N	N	N	Y	Y	Y	Y	7

1—Eligibility criteria; 2—random allocation; 3—concealed allocation; 4—baseline comparability; 5—blinding of subjects; 6—blinding of therapists; 7—blinding of assessors; 8—adequate follow-up; 9—intention-to-treat analysis; 10—between-group statistical comparisons; 11—point estimates and measures of variability. Y—criterion met; N—criterion not met; ∑—total score awarded.

**Table 3:** Studies included in the qualitative analysis.

Study	Age [Years]	Number and Groups	Duration [Weeks] Sessions [Per Week]	Program (Type, Intensity Frequency, Training Duration)		Measured Outcomes			Results	
				E Group	C Group	Body Composition	Physical Fitness	VO^2^_max_		
**Maha et al. (2015)** [31]	**__**	N-20 HIIT-10, MCT-10	6 week 3 sessions	HIIT, 85–95% HRmax	MCT, 60–70% HRmax	__	__	VO^2^_max_ VT 3000-m race PPO MPO	HIIT: VO^2^_max_ ↑ * VT↑ 3000 mr↑ * PPO ↑ * MPO↑	MCT: VO^2^_max_ ↑ * VT↑ 3000 mr↓ PPO ↓ MPO↓
**Sanchez-Sanchez et al. (2018)** [32]	17.2 ± 1.1	N-12 HITCOD1–6, HITCOD3–6	6 weeks 2 sessions	COD1- HIIT (1 COD), 90%, (+6 regular practices), 2 × 6 min	COD3- HIIT (3 CODs), 90%, 2 × per week (+6 regular practices), 2 × 6 min	__	V-cut RSA	VIFT	HITCOD1 V-cut ↑ RSA- ↑ VIFT- ↑	HITCOD3 MAT 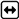 V-cut ↑ * RSA- ↑ * VIFT- ↑ *
**Aschendorf** **et al. (2019)** [33]	15.1 ± 1.1	N-24, TG-11, CG-13	5 weeks 2 sessions	Specific basketball practices HIIT, 90–90% HRmax; 25 min	Regular field practices	BF FFM	CMJ CMJa SJ COD	YYIR1 Shuttle run 20 m	TG: COD180 ↑ CMJ 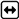 CMJa 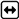 SJ 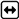 YYIR ↑ BF 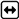 FFM 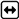	CG: COD180 ↓ * CMJ 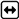 CMJa 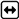 SJ 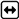 YYIR1 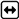 BF 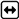 FFM 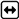
**Zeng et al. (2022)** [34]	19.9 ± 1.1	N-19 SSG-9, HITCOD -10	4 weeks 3 sessions	HIITCOD: 3 × (6 min od 15″-15″ 90% VIFT)	SSG: 3 × (2 × 2 min 45), 2 min passive rest	__	MAT CMJ SAT RSA	30-15 IFT Shuttle run 20 m	SSG: 30-15 IFT↓ RSA↑ MAT↑ * CMJ↓	HIIT: 30-15 IFT↓ RSA↑ MAT↑ CMJ↓
**Rodríguez-Fernández, et al. (2023)** [35]	17.9 ± 0.6	N-16 C-HIIT-8 N-HIIT-8	6 weeks 2 sessions	HIIT 30 s/30 s (passive) 90% 40 m 2 × 12 min [3 min]	HIIT 15 s/15 s (passive) 100% 40 m 2 × 6 min (6 min)	__	__	30-15 VIFT RSA	C-HIIT: 30-15 IFT↑ * RSA 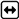	N-HIIT: 30-15 IFT ↑ RSA 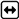
**Apaydın, et al. (2023)** [36]	15.7 ± 0.93	N-20	8 weeks 2 sessions	90–95% HRmax	Regular field practices	BMI	VJ 10 m 20 m	__	HIIT BMI 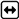 VJ↑ 10m↑ * 20m↑ *	CG: BMI 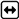 VJ 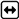 10m 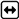 20m↑ *
**Mourgan, et al. (2024)** [37]	15.1 ± 1.1	N-24 TG-12 CG-12	5 weeks 2 h	HIIT 90–95% HRmax; 25 min, 4 min HIIT 3 min rest	Regular field practices	__	CMJ SJ COD20	VO^2^_max_ VCO_2_ VEGF	TG: CMJ↑ SJ↑ VO^2^_max_ ↑ * VCO_2_↑ * VEGF ↑ COD20↑ *	CG:
**Haghighi, et al. (2024)** [38]	14–16	N-24 HIIT-8 PT-8 CG-8	6 weeks 2 sessions	HIIT: 90–95% HRmax, 2 × per week, 30–60 min	Plyometric sessions, 2 × per week, 30–60 min	__	CMJ SJ 20m	RSA VO^2^_max_ 30-15 IFT	PT	HIIT
**Fang et al. (2024)** [39]	23.1 ± 1.5	N-20 CG-10 TG-10	6 weeks 2 sessions	SIT 3 sets of 10 repetitions	Regular field practices	__	VJ 20m Illionois *T* test	YYIR1 VO^2^_max_	VJ↑ * 20m↑ * Illionois↑ *T* test↑ * YYIR1↑ * VO2max↑ *	VJ 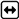 20m 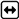 Illionois 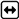 *T* test 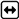 YYIR1 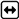 VO2max 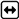

N—number of participants; ↑—statistically significant increase at *p* < 0.05; ↑ *—statistically significant increase at *p* < 0.01; ↓—statistically significant decrease at *p* < 0.05; ↓ *—statistically significant decrease at *p* < 0.01; 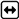
—no statistically significant change; E group—experimental group; TG—training group; CG—control group; HIIT—high-intensity interval training; MCT—medium continuous training; VO_2max_—maximal oxygen uptake; PPO—peak power output; MPO—mean power output; V-cut—25 m maximal running test with four directional changes (around four cones); RST—repeated sprint test; VIFT—velocity at the last completed stage of the intermittent fitness test; MAT—modified agility test; CMJ—countermovement jump; CMJa—countermovement jump with arm swing; SJ—squat jump; YYIR1—Yo-Yo intermittent recovery test level 1; COD—change-of-direction test; 20/40 m Shuttle Run—test for evaluating agility; BF—body fat percentage; FFM—fat-free mass; COD180—change-of-direction test with a 180° turn; VT—ventilatory threshold; SAT—shooting accuracy test; RSA—repeated sprint ability; 30-15 IFT—30-15 intermittent fitness test; 3000 m race—3000 mr; SSG—small-sided games; HR—heart rate *.

## Data Availability

No new data were created or analyzed in this study.
